# Classification of Lung Adenocarcinoma Based on Immune Checkpoint and Screening of Related Genes

**DOI:** 10.1155/2021/5512325

**Published:** 2021-07-27

**Authors:** Ting Zhou, Ping Yang, Sanyuan Tang, Zhongshan Zhu, Xiaobing Li, Zhou Yang, Ruoxia Wu, Xuefei Tian, Liang Li

**Affiliations:** ^1^Department of Oncology, Brain Hospital of Hunan Province, Changsha 410007, Hunan Province, China; ^2^Department of Internal Medicine, College of Integrated Chinese and Western Medicine, Hunan University of Chinese Medicine, Changsha, 410208, Hunan Province, China; ^3^Department of Psychiatry, Brain Hospital of Hunan Province, Clinical Medical College of Hunan University of Chinese Medicine, Changsha 410007, China; ^4^Hunan Key Laboratory of TCM Prescription and Syndromes Translational Medicine, Hunan University of Chinese Medicine, Changsha, 410208, Hunan Province, China; ^5^Provincial Key Laboratory of TCM Diagnostics, Hunan University of Chinese Medicine, Changsha 410208, Hunan Province, China

## Abstract

**Aims:**

Lung adenocarcinoma (LUAD) cells could escape from the monitoring of immune cells and metastasize rapidly through immune escape. Therefore, we aimed to develop a method to predict the prognosis of LUAD patients based on immune checkpoints and their associated genes, thus providing guidance for LUAD treatment.

**Methods:**

Gene sequencing data were downloaded from the Cancer Genome Atlas (TCGA) and analyzed by R software and R Bioconductor software package. Based on immune checkpoint genes, kmdist clustering in ConsensusClusterPlus R software package was utilized to classify LUAD. CIBERSORT was used to quantify the abundance of immune cells in LUAD samples. LM22 signature was performed to distinguish 22 phenotypes of human infiltrating immune cells. Gene set variation analysis (GSVA) was performed on immune checkpoint cluster and immune checkpoint score using GSVA R software package. The risk score was calculated by LASSO regression coefficient. Gene Ontology (GO), Hallmark, and Kyoto Encyclopedia of Genes and Genomes (KEGG) analysis were performed. PROC was performed to generate the ROC curve and calculate the area under the curve (AUC).

**Results:**

According to the immune checkpoint, LUAD was classified into clusters 1 and 2. Survival rate, immune infiltration patterns, TMB, and immune score were significantly different between the two clusters. Functional prediction showed that the functions of cluster 1 focused on apoptosis, JAK/STAT signaling pathway, TNF-*α*/NF*κ*B signaling pathway, and STAT5 signaling pathway. The risk score model was constructed based on nine genes associated with immune checkpoints. Survival analysis and ROC analysis showed that patients with high-risk score had poor prognosis. The risk score was significantly correlated with cancer status (with tumor), male proportion, status, tobacco intake, and cancer stage. With the increase of the risk score, the enrichment of 22 biological functions increased, such as *p*53 signaling pathway. The signature was verified in IMvigor immunotherapy dataset with excellent diagnostic accuracy.

**Conclusion:**

We established a nine-gene signature based on immune checkpoints, which may contribute to the diagnosis, prognosis, and clinical treatment of LUAD.

## 1. Background

Lung adenocarcinoma (LUAD) accounts for 40% of lung cancer patients and is one of the main subtypes of lung cancer [1]. LUAD is characterized by diffuse metastasis and poor prognosis. The survival rate of patients is less than five years [2]. Because of the high resistance of LUAD to radiotherapy and chemotherapy [3], it is urgent to look for treatment from other directions.

Recently, immunotherapy has become an emerging strategy for cancer treatment [4]. Immune system is involved in the growth and metastasis of cancer cells. For example, cancer cell antigens are recognized by dendritic cells and other antigen presenting cells, which promote the maturation of the corresponding cytotoxic CD8+ T lymphocytes [5, 6]. However, any mistakes in this process might lead to the failure of antigen presenting cells to present antigen normally. It is well known that the complex tumor microenvironment is an important reason for the failure of immunotherapy. As T cells failure to reach the tumor area, the immune system was unable to recognize and remove cancer cells [7]. Wu et al. developed a scoring system based on the expression of genes related to tumor metabolism in LUAD that is suitable not only for predicting prognosis in patients with LUAD but also for predicting LUAD response to checkpoint immunotherapy [8]. Currently, based on the blockade of immune checkpoint, tumor immunotherapy has made a breakthrough progress.

Immune checkpoints were defined as ligand receptor pairs that inhibit or stimulate immune responses [9]. Tumor cells evaded the attack of immune cells by activating immune checkpoints [10]. Currently, some drugs have been developed to block the immune checkpoint. The combination of ipilimumab and placebo can prevent CTLA-4 from binding to its ligand, thus blocking the immune checkpoint [11]. High expression of PD-1 could inhibit the function of T cells and promote the immune escape of tumor cells [12, 13]. Nivolumab is an antibody against PD-1, which could play an antitumor role in clinical trials. However, patients have side effects of loss of appetite and diarrhea [14]. Therefore, to develop better targeted drugs, we need to find more representative immune checkpoints.

TCGA database is often utilized to predict reliable biomarkers [15]. Based on the analysis of TCGA dataset, we classified LUAD according to immune checkpoint genes and detected the clinical characteristics of patients after classification. Simultaneously, based on immune checkpoint-related genes, we established a risk score model and analyzed its correlation with the clinical characteristics and signaling pathways of LUAD samples.

## 2. Materials and Methods

### 2.1. LUAD Dataset and Preprocessing

The Cancer Genome Atlas (TCGA) dataset was downloaded from UCSC Xena (https://xenabrowser.net/). RNA sequencing (RNA-seq) data was downloaded from TCGA data portal. Then, the fragments per kilobase million (FPKM) were converted to transcripts per million (TPM). The microarray dataset GSE68465 was downloaded from Gene-Expression Omnibus (GEO; https://www.ncbi.nlm.nih.gov/geo/) as an external validation set. The raw data from the microarray dataset was generated by Affymetrix. Then, the RMA algorithm in Affy package was applied to process the original data from Affymetrix for quantile normalization and background correction. All data were analyzed by R software (version 3.6.1) and R Bioconductor software package.

### 2.2. Identification of Related Classification of LUAD Immune Checkpoint

Sixty-five immune checkpoint genes were obtained for subsequent clustering. The filtering procedure was performed. The kmdist clustering method in ConsensuClusterPlus R software package was utilized to classify LUAD by immune checkpoint genes [16], so as to determine the patterns related to the immune checkpoint, and the patients were grouped for further analysis.

### 2.3. Estimation of Immune Infiltration

CIBERSORT algorithm was used to quantify the abundance of immune cells in LUAD samples using Leukocyte signature matrix (LM22) as an eigenmatrix [17], since LM22 gene signature could sensitively and specifically distinguish 22 phenotypes of human infiltrated immune cells. The algorithm was run with an LM22 signature and 1000 permutations. Gene expression profiles have been uploaded to CIBERSORT web portal (http://cibersort.stanford.edu/).

### 2.4. Pathway Analysis

All gene sets were downloaded from MSigDB database [18]. Gene Set Variation Analysis (GSVA) R software package was utilized to analyze the GSVA of immune checkpoint cluster and immune checkpoint score [19], including Gene Ontology Biological Process (GO BP), Kyoto Encyclopedia of Genes and Genomes (KEGG), and Hallmark gene sets. The selection criteria of immune checkpoint cluster related pathway was based on the corrected *p* < 0.05. The selection criteria of immune checkpoint score related pathway was based on the correlation analysis *p* < 0.05.

### 2.5. Establishment of Immune Checkpoint Score

Limma package in R software was performed to identify differential genes (DEG) associated with two immune checkpoint-related patterns [20]. The corrected *p* value <0.05 and | logFC | < 1 were set as the significance criteria to determine DEG in immune checkpoint subtypes. Univariate Cox regression analysis was utilized to certificate the representative DEG, and then, random forest in machine learning method of caret package was used to reduce gene dimension. The TCGA dataset was randomly divided into two parts, each of them containing 250 samples. One part of the data was performed to train the model, and the other part and the total data were used to verify the model. In the training set, the dimensionality reduced genes were utilized for single-factor Cox analysis to screen meaningful genes, and then, the highest lambda value (“min” lambda) was selected through 1000 cross-validation in LASSO method. We obtained a group of prognostic genes and their LASSO regression coefficients. Risk score was the sum of the expression value of genes screened by LASSO regression coefficient.

Risk score = (−0.0044)*∗*

CCR7 (gene expression level) + 0.0323*∗*

CPS1 + (−0.1006)*∗*

LILRB1 + 0.1460*∗*

GOS2 + (−0.2027)*∗*

HLA-DOB + (−0.2116)*∗*

CCR2 + (−0.1181)*∗*

CLEC7A + 0.3002*∗*

BIRC3 + (−0.0286)*∗*

CD1E.

### 2.6. Survival Curve and ROC Analysis

The pROC package was used to generate receiver operating characteristic (ROC) curve and calculate area under curve (AUC) [21]. Kaplan–Meier was utilized to generate and visualize survival curves of subgroups. The statistical significance of the differences in each dataset was determined by log rank test. All survival curves were generated by R package survminer. All heatmaps were generated based on pheatmap. All statistical analysis were analyzed in R (https://www.r-project.org/, version 3.6.1).

### 2.7. Statistical Analysis

The Shapiro–Wilk test was utilized to assess the normality of variables. For normally distributed variables, unpaired Student's *t*-test was used to compare the differences between the two groups. Wilcoxon test was utilized to compare variables with nonnormal distribution. For multiple groups, ANOVA was performed as the parametric method to compare the mean value, while Kruskal–Wallis test was used as the nonparametric method. Pearson correlation and distance correlation analysis were used to calculate the correlation coefficient. According to the risk score of dichotomy, patients were divided into high- or low-risk score of each dataset. Ggplot2 was used of visualization in the R program. In the analysis of differentially expressed genes, we utilized Benjamini-Hochberg, which transformed *p* value into FDR to identify important genes. All the tests were two-sided, and *p* values <0.05 were considered statistically significant.

## 3. Results

### 3.1. Distribution of Immune Checkpoints after Gene Clustering

**Figure 1:**
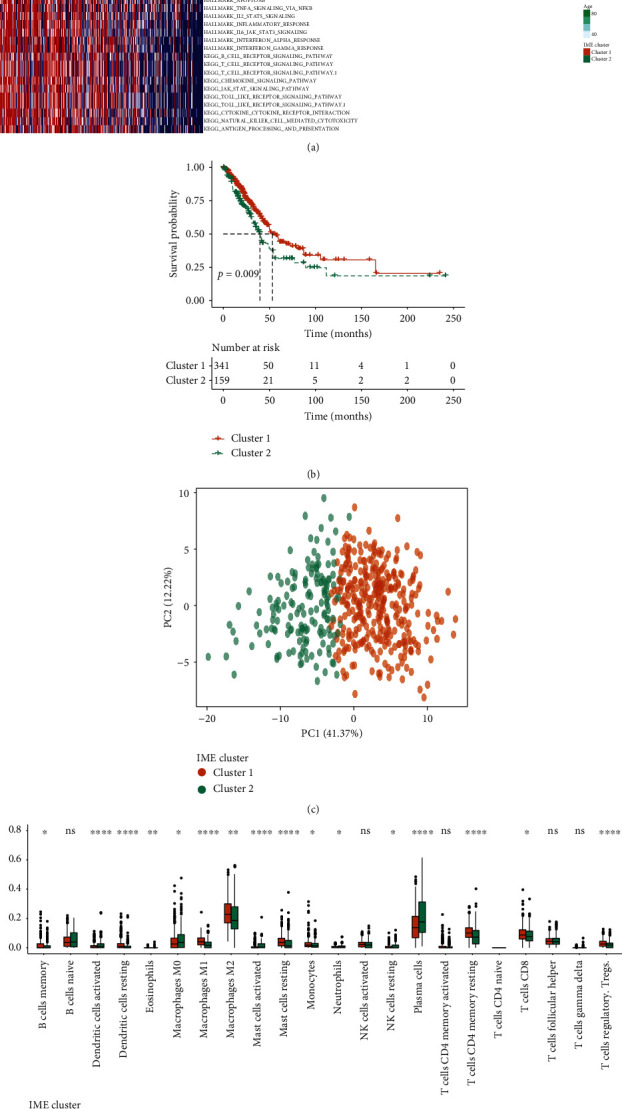
The characteristics of clusters 1 and 2. (a) Correlation between immune checkpoint-related gene expression and clinical characteristics in two clusters. (b) Survival analysis of clusters 1 and 2. (c) PCA analysis of clusters 1 and 2. (d) Comparison of the distribution of immune cells in clusters 1 and 2.

We clustered LUAD by immune checkpoint. The results showed that the boundaries of LUAD clustering into two categories were clear (Figure S1A), while the boundaries of lung adenocarcinoma clustering into three or four categories were fuzzy (Figures S1B and S1C). Consistency cluster analysis (consensus CDF) was used for further analysis, and the best classification was defined as the one with the smallest slope of curve decline in the abscissa range of 0.1–0.9. The results showed that when LUAD was divided into two categories, clusters 1 and 2, according to immune checkpoints, the descending slope of curve was the smallest. The correlation between immune checkpoint-related gene expression and clinical characteristics in the two clusters was shown in Figure 1(a). Most genes were highly expressed in cluster 1 and low expressed in cluster 2. We also found that the expression of molecules related to antigen presentation (HLA-C, HLA-B), ligands (CXCL5, CXCL10), receptors (ADORA2A, BTLA), inhibitors (btn3a1, btn3a2), activators (CD28, CD80), and cell adhesion (ICAM1, ITGB2) in cluster 1 was significantly higher than that in cluster 2. Other proteins involved in immune regulation, such as IDO1, GZMA, and PRF1, were also highly expressed in cluster 1 compared with cluster 2 (Figure S2). We used the survival curve to analyze the survival difference between clusters 1 and 2. It was found that the survival rate of patients in cluster 2 decreased faster than that in cluster 1 over time. This indicated that the prognosis of patients in cluster 2 was worse than that in cluster 1. PCA image showed that clusters 1 and 2 can be separated (Figure 1(c)), which proved that the classification of LUAD was meaningful. Different types of immune cells were distributed differently in the two clusters (Figure 1(d)). For example, the distribution of B cells memory, dendritic cells resting, macrophages M1 and M2, T cells CD4 memory resting, and T cells CD8 in cluster 1 were more abundant than those in cluster 2. While the distribution of plasma cells, dendritic cells activated, macrophages M0, mast cells activated, and NK cells resting in cluster 2 were contrary.

### 3.2. Functional Analysis of Checkpoints in Clusters 1 and 2

**Figure 2:**
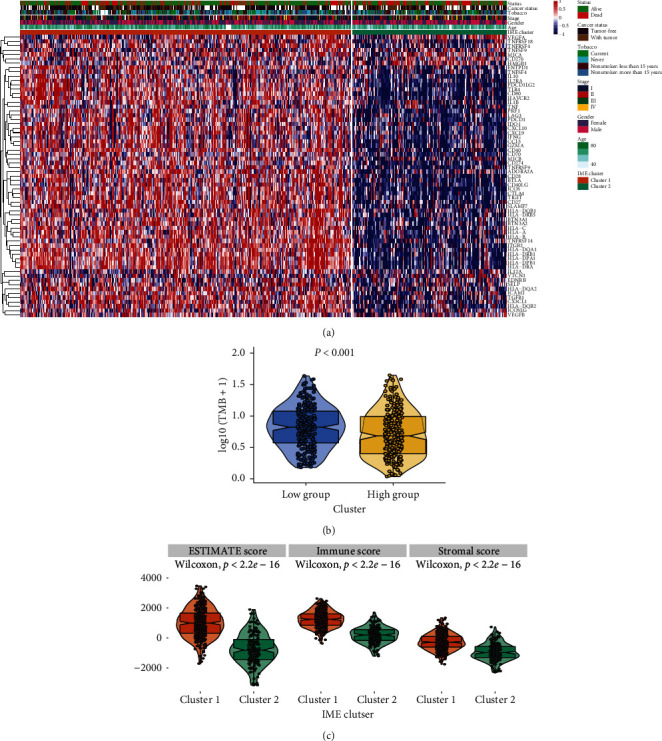
Functional analysis and immune score of clusters 1 and 2. (a) Functional analysis of the two clusters was based on GO enrichment, Hallmark, and KEGG pathway. (b) Tumor mutation burden of clusters 1 and 2. (c) Immune infiltration assessment in clusters 1 and 2.

We performed GO analysis, Hallmark, and KEGG pathway to analyze the function of immune checkpoint-related genes in clusters 1 and 2 (Figure 2(a)). GO analysis results showed that mitochondrial RNA catabolic process was significantly enriched in cluster 2. The related genes of cluster 1 were mainly involved in macrophage proliferation, positive regulation of inflammatory response to antigenic stimulus, CD8 positive alpha beta T cell activation, and microglial cell activation. Hallmark results showed that cluster 2 genes were involved in Myc targets V2, G2/M checkpoint, and DNA repair. The genes in cluster 1 were mainly involved in apoptosis, TNFa signaling via NFkB, and IL2 STAT5 signaling. In addition, KEGG pathway showed that, compared with cluster 2, genes in cluster 1 were mainly enriched in T and B cell receptor signaling pathway, chemokine signaling pathway, JAK/STAT signaling pathway, and toll-like receptor signaling pathway. These results indicated that immune-related pathway scores were significantly higher in cluster 1 than in cluster 2. Then, we examined the tumor mutation burden (TMB) of clusters 1 and 2, and the results showed that cluster 1 was higher than cluster 2 (Figure 2(b)). Finally, we found that the ESTIMTE score, immune score, and stromal score of cluster 1 were significantly higher than those of cluster 2 (*p* < 2.22*e* − 16) (Figure 2(c)). These results suggested that the survival and prognosis of cluster 1 patients were better than those of cluster 2 patients.

### 3.3. Screening of Genes in Clusters 1 and 2 and Establishment of Prognosis Model

**Figure 3:**
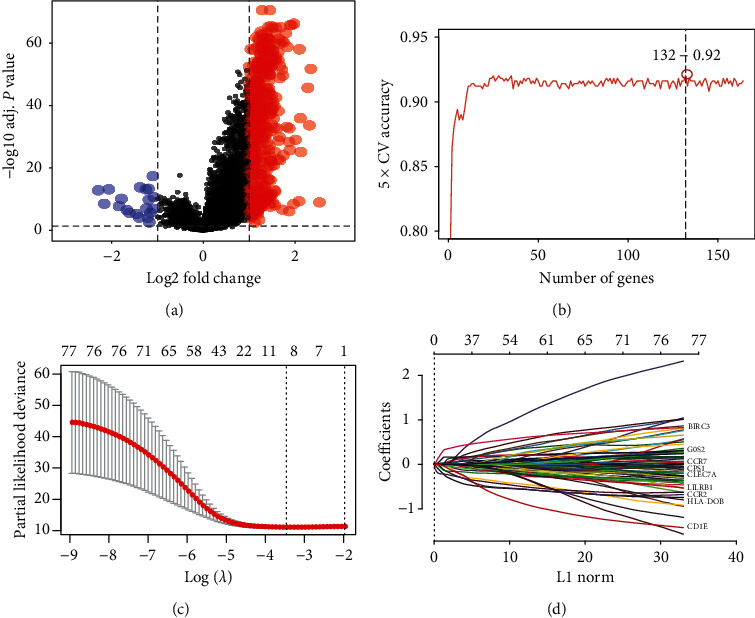
Screening of immune checkpoint-related genes. (a) Differential expressed genes between the two clusters. (b) The number of genes obtained by random forest dimensionality reduction. (c) Partial likelihood deviance coefficient profiles of 77 immune checkpoint genes via 10-fold cross-validation. (d) Least absolute shrinkage and selection operator (LASSO) profiles of the selected genes.

The total number of genes differing between the two clusters was 552 (Figure 3(a)). Later, we screened 164 genes by univariate analysis in TCGA dataset and further obtained 132 genes by random forest dimensionality reduction (Figure 3(b)). Through single factor analysis of 132 genes in the modeling set, 77 genes were obtained (Figure 3(c)), Subsequently, nine genes, including BIRC3, G0S2, CCR7, CPS1, CLEC7A, LILRB1, CCR2, HLA-DOB, and CD1E, were screened by LASSO method (Figure 3(d)). These nine genes were divided into high-risk group and low-risk group, to establish the risk score formula: risk score = (−0.0044)*∗*

CCR7 (gene expression level) + 0.0323*∗*

CPS1 + (−0.1006)*∗*

LILRB1 + 0.1460*∗*

GOS2 + (−0.2027)*∗*

HLA-DOB + (−0.2116)*∗*

CCR2 + (−0.1181)*∗*

CLEC7A + 0.3002*∗*

BIRC3 + (−0.0286)*∗*

**Figure 4:**
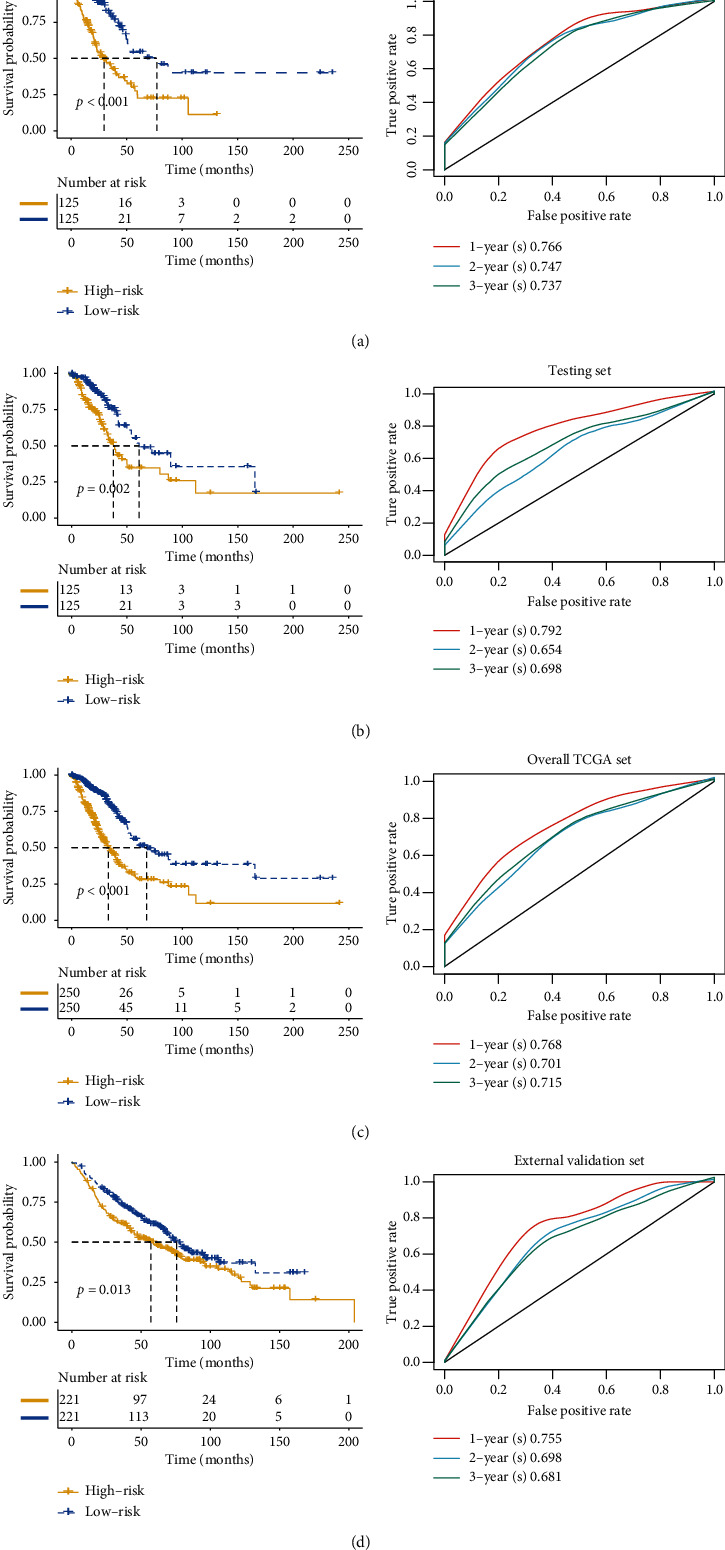
Survival curve analysis and ROC analysis of the nine-gene signature. (a) Survival analysis of two risk groups and ROC analysis of the nine-gene signature in training set. (b) Survival curve of two risk groups and ROC curve of the nine-gene signature in testing set. (c) Survival status of two risk groups and ROC analysis of the nine-gene signature in overall TCGA set. (d) Survival analysis and ROC curve of the nine-gene signature in the external validation set.

CD1E. Then, we used the risk score model to analyze the survival rate of patients with these nine genes in the training set (Figure 4(a)), testing set (Figure 4(b)), overall TCGA set (Figure 4(c)), and external validation set (Figure 4(d)). Survival analysis showed that patients with high-risk score had a poor prognosis (*p* < 0.05). Time-dependent ROC analysis showed that the one-year AUC reached 0.755 in all datasets. It suggested that the efficacy of the model was satisfactory. To ensure the robustness of the model, we verified the LUAD model with IMvigor immunotherapy dataset, as shown in Figure S3. The survival rate of the high-risk group was lower than that of the low-risk group. It demonstrated the accuracy of our model in predicting prognosis.

### 3.4. Analysis of the Relationship between Risk Score, Clinical Characteristics, and Signaling Pathways

**Figure 5:**
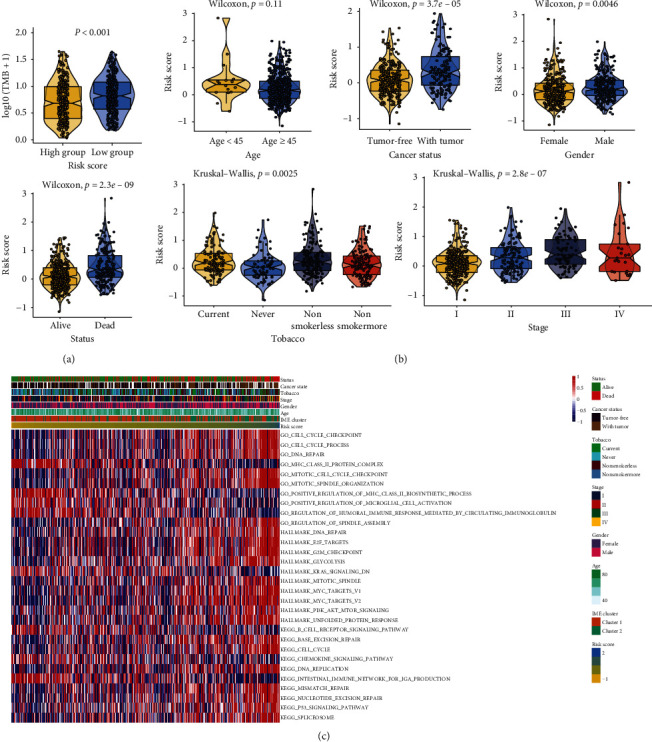
Analysis of the relationship between risk score, clinical characteristics, and signaling pathways. (a) Comparison of TMB between the two risk groups. (b) The relationship between risk score model and clinical characteristics, including age, cancer status, gender, status, tobacco, and stage. (c) Function prediction of GO, Hallmark, and KEGG pathways based on risk score model.

According to the risk score of these nine genes, we detected the changes of different clinical characteristics of the patients. The results showed that the TMB value of the low-risk score group was higher than that of the high-risk score group (Figure 5(a)). Then, we evaluated the relationship between risk score and clinical characteristics. There was no significant difference between high-risk score and low-risk score in age (*p*=0.11). The risk score exhibited statistical differences in cancer status (tumor-free, with tumor), gender (female, male), status (alive, dead), tobacco (current smoker, never smoke, nonsmoker: less than 15 years, and nonsmoker: more than 15 years, and stage (I, II, III, IV) (*p* < 0.05) (Figure 5(b)). We also evaluated the relationship between the risk score and biological function (Figure 5(c)). GO analysis showed that the expression of cell cycle related pathways and DNA repair increased as the risk score increased gradually, while the expression of MHC type II protein decreased. Hallmark results showed that the expression of E2F target, G2/M checkpoint, and Myc target increased, and the expression of KRAS signaling pathway decreased, when the risk score gradually increased. KEGG pathway showed that seven pathways, including base excision repair, cell cycle, and DNA replication, were positively correlated with the risk score. B cell receptor signaling pathway, chemokine signaling pathway, and intestinal immune network for IgA production were negatively correlated with the risk score.

## 4. Discussion

LUAD has the characteristics of poor prognosis and rapid metastasis, which leads to high mortality. Many studies have shown that tumor development is controlled by the patient's immune system, which has important implications for patient prognosis and response to drug therapy [22]. Therefore, we classified LUAD according to immune checkpoint. After classification, we found that the expression of molecules related to antigen presentation, ligands, receptors, inhibitors, activators, and cell adhesion in cluster 1 was significantly higher than that in cluster 2. Simultaneously, the survival rate of patients in cluster 1 was higher than that in cluster 2. The results of immunocyte distribution showed that there were more immunocytes in cluster 1, but the dendritic cells were dormant. In cluster 2, the dendritic cells were activated, but the cells with lethality were less. Thus, we suspect that there is something wrong with the antigen presentation or immune checkpoint.

In our study, the results of pathway prediction showed that the expression of immune cell activation related pathways in cluster 1 was significantly higher than that in cluster 2. Therefore, we speculated that this may lead to more immune cells in cluster 1 than in cluster 2. In addition, the function of checkpoint in cluster 1 was also enriched in apoptosis, JAK/STAT signaling pathway, TNF-*α*/NF *κ* B signaling pathway, and STAT5 signaling pathway. The continuous activation of JAK/STAT signaling pathway was closely related to many immune and inflammatory diseases, which was closely related to the recognition of tumor cells by immune cells and the immune escape process of tumor [23]. Taking NK cells as an example, the early maturation, development, and functional activation of NK cells were strictly regulated by cytokines of JAK/STAT pathway [24]. IL-2 and IL-15 promoted NK cell homeostasis, proliferation, and function through STAT5 transduction [25] NF*κ*B signaling pathway was also involved in the regulation of JAK/STAT pathway on immune cells [18].

We screened the immune checkpoint-related genes, BIRC3, G0S2, CCR7, CPS1, CLEC7A, LILRB1, CCR2, HLA-DOB, and CD1E, and detected the effect of these genes on the survival rate of patients. When the risk index of these genes increased, the survival rate of patients decreased. After literature search, it was found that these genes can be used as therapeutic targets for cancer. For example, birc3 was associated with chemoimmunotherapy resistance, and its inactivation also affects tumor cells that depend on NF*κ*B pathway to survive [26]. Wang et al. found that hypoxia could induce birc3 expression through HIF1 alpha signal transduction mechanism in glioblastoma cells [27]. The sensitivity of G0/G1 transition gene 2 (G0S2) breast cancer cells to tamoxifen was relatively increased, which makes G0S2 an antitumor breast cancer target and biomarker of recurrence and therapeutic response [28, 29]. CCR7 and CD163 could be used as markers of macrophage polarization in lung cancer microenvironment [30]. We speculated that these genes may regulate the activity of immune cells in tumor microenvironment. After that, we will further explore the specific regulatory role of these genes on immune cells. We verified the clinical changes and related pathway changes of LUAD patients after the rise of the risk score of nine genes. We found that the high-risk group showed lower TMB. Ouyang et.al. showed that TMB was related to the immunotherapy response of colon adenocarcinoma. In the group with high TMB level, higher infiltration of CD8+ T cells, CD4+ T cells, and other immune cells could be observed in the cancer tissue [31]. This suggests that when the risk score is increased, the distribution of immune cells in the tumor may decrease correspondingly, leading to immune escape of tumor cells. In our results, the risk score was positively correlated with KARs pathway activation. KARS gene was a proto oncogene. Smoking could cause KARS mutation and carcinogenesis. Studies have shown that curcumin can be used as a sensitizer to overcome the resistance of NSCLC patients with wild-type EGFR and/or KRAS mutations [32]. However, it is worth noting that *p*53 is a tumor suppressor protein, and gene therapy has been found to increase the expression of *p*53 [33]. The increased expression of *p*53 pathway in our experiment still needs further study.

Current radiotherapy and chemotherapy are difficult to meet the needs of the treatment of LUAD. Currently, bioinformatics technology has been used to screen biomarkers of LUAD, which was more targeted for the treatment of the disease. Song et al. established a gene model consisting of 30 immune-related genes to predict the prognosis of LUAD [34]. In this study, we established a nine-gene prognostic model for LUAD based on immune checkpoints. The IMvigor dataset verified its prognostic value. From the perspective of clinical application, nine key genes associated with immune checkpoints identified in this study may be easier to be detected in clinical practice.

There are still some limitations in this study. First, our data comes from a public database, and potential selection bias cannot be ruled out. Second, the robustness of our results needs to be further validated in prospective studies. In addition, as there are not enough clinical samples, we have not tested this model in clinical trials. Therefore, we will clarify the biological function of the predicted genes on the basis of the present work in combination with experimental verification.

## 5. Conclusion

In conclusion, we identified a nine-gene signature based on immune checkpoints that may be used to aid prognostic analysis in patients with LUAD. Our study may provide several valuable molecular targets for immunotherapy of LUAD.

## Data Availability

The data of this study were taken from the TCGA and GEO databases.

## Supplementary Materials

Supplementary Materials Figure S1: cluster analysis of lung adenocarcinoma. (A) Consensus matrix, *K* = 2. (B) Consensus matrix, *K* = 3. (C) Consensus matrix, *K* = 4. (D) Empirical CDFs corresponding to the entries of consensus matrix for *K* = 2, 3,… and 10. Figure S2: the expression of immune process related proteins in clusters 1 and 2. (A) Antigen present. (B) Ligand. (C) Receptor. (D) Coinhibitor. (E) Costimulator. (F) Cell adhesion. (G) Others. Figure S3: Kaplan–Meier survival curves showed the effectiveness of the risk prediction model in IMvigor immunotherapy dataset.
