# Supplementary material for: Classification of Lung Adenocarcinoma Based on Immune Checkpoint and Screening of Related Genes
Source: J Oncol. 2021 Jul 27;2021:5512325. doi: 10.1155/2021/5512325 (PMC8337117; doi:10.1155/2021/5512325)
Supplement: Supplementary Materials — Figure S1: cluster analysis of lung adenocarcinoma. (A) Consensus matrix, K = 2. (B) Consensus matrix, K = 3. (C) Consensus matrix, K = 4. (D) Empirical CDFs corresponding to the entries of consensus matrix for K = 2, 3,… and 10. Figure S2: the expression of immune process related proteins in clusters 1 and 2. (A) Antigen present. (B) Ligand. (C) Receptor. (D) Coinhibitor. (E) Costimulator. (F) Cell adhesion. (G) Others. Figure S3: Kaplan–Meier survival curves showed the effectiveness of the risk prediction model in IMvigor immunotherapy dataset. [file 5512325.f1.zip › 5512325.f1/supplementary description.docx]

**Supplementary Material:**

Figure S1. Cluster analysis of lung adenocarcinoma. (A) consensus matrix, K=2. (B) consensus matrix, K=3. (C) consensus matrix, K=4. (D) empirical CDFs corresponding to the entries of consensus matrix for K = 2, 3, ......and 10.

Figure S2. The expression of immune process related proteins in cluster 1 and cluster 2. (A) Antigen present. (B) Ligand. (C) Receptor. (D) Co-inhibit. (E) Co-stimulator. (F) Cell adhesion. (G) Other

Figure S3. Kaplan-Meier survival curves showed the effectiveness of the risk prediction model in ImVigor immunotherapy dataset.
